# Exercise-Induced Biomarker Modulation in Sarcopenia: From Inflamm-Aging to Muscle Regeneration

**DOI:** 10.3390/sports13120444

**Published:** 2025-12-09

**Authors:** Federica Marmondi, Vittoria Ferrando, Luca Filipas, Roberto Codella, Piero Ruggeri, Antonio La Torre, Emanuela Luisa Faelli, Matteo Bonato

**Affiliations:** 1 Department of Neuroscience, Rehabilitation, Ophthalmology, Genetics and Maternal Child Health, University of Genoa, 16132 Genoa, Italy; 2 Centro Polifunzionale di Scienze Motorie, University of Genoa, 16126 Genoa, Italy; vittoria.ferrando@unige.it (V.F.); ruggeri@unige.it (P.R.); emanuela.faelli@unige.it (E.L.F.); 3 Department of Experimental Medicine, Section of Human Physiology, University of Genoa, 16132 Genoa, Italy; 4 Department of Biomedical Sciences for Health, Università degli Studi di Milano, 20122 Milan, Italy; luca.filipas@unimi.it (L.F.); roberto.codella@unimi.it (R.C.); antonio.latorre@unimi.it (A.L.T.); matteo.bonato@unimi.it (M.B.); 5 Department of Endocrinology, Nutrition and Metabolic Diseases, IRCCS MultiMedica, 20138 Milan, Italy; 6 Laboratory of Movement and Sport Sciences (LaMSS), IRCCS Istituto Ortopedico Galeazzi, Via Cristina da Belgioioso 173, 20157 Milan, Italy

**Keywords:** inflamm-aging, exerkines, muscle wasting

## Abstract

Sarcopenia is a progressive, age-related loss of skeletal muscle mass, strength, and function, strongly associated with frailty, disability, and chronic disease. Its pathogenesis involves chronic low-grade inflammation, hormonal imbalance, and impaired anabolic signaling, making biomarkers essential for diagnosis, prognosis, and intervention monitoring. This review systematically analyzes randomized controlled trials (RCTs) evaluating the impact of physical exercise on biomarkers relevant to sarcopenia. Exercise modulates both pro-inflammatory markers (e.g., IL-6, TNF-α, CRP) and anti-inflammatory cytokines (e.g., IL-10, IL-15), while also affecting growth factors like IGF-1, myostatin, and follistatin. These changes support muscle anabolism, reduce catabolic signaling, and improve physical performance. In addition, we highlight a growing class of emerging exerkines, including irisin, apelin, beta-aminoisobutyric acid (BAIBA), decorin, brain-derived neurotrophic factor (BDNF), and meteorin-like factor (Metrnl). These molecules exhibit promising roles in mitochondrial health, lipid metabolism, muscle regeneration, and immune modulation, key processes in combating inflamm-aging and sarcopenic decline. Despite encouraging findings, biomarker responses remain heterogeneous across studies, limiting translational application. The integration of biomarker profiling with exercise prescription holds the potential to personalize interventions and guide precision medicine approaches in sarcopenia management. Future large-scale, standardized trials are needed to validate these biomarkers and optimize exercise protocols for aging populations.

## 1. Sarcopenia: From Pathophysiology to Biomarkers and Exercise Modulation

Sarcopenia is a progressive and generalized loss of skeletal muscle mass, strength, and function, primarily associated with aging but also exacerbated by chronic diseases such as type 2 diabetes, cardiovascular disease, and cancer, with profound implications for mobility, independence, and quality of life [1,2,3]. Initially defined by Rosenberg in 1989 as a loss of muscle mass, the condition has been redefined to include not only quantitative but also qualitative impairments, encompassing declines in strength and physical performance [4,5,6]. Based on criteria such as low muscle strength, reduced muscle quantity, and impaired physical performance, sarcopenia is now stratified into probable, confirmed, and severe stages [1,7]. Prevalence increases with age, affecting 10–27% of adults over 60 and up to 50% beyond the age of 80 [8,9], with even higher rates reported in hospitalized and institutionalized populations [1,10]. Sarcopenia is associated with increased risks of frailty, falls, fractures, metabolic dysfunction, hospitalization, and mortality, contributing to a growing burden on healthcare systems [11,12,13,14]. While no pharmacological treatments have yet been approved, lifestyle-based interventions remain the cornerstone of prevention and management. Resistance and multicomponent exercise training have shown strong evidence in preserving muscle mass, strength, and mitochondrial function, while nutritional strategies, including protein and vitamin D supplementation, further support muscle maintenance [15,16,17,18,19]. Aging is accompanied by complex changes in muscle tissue, including fiber atrophy, fatty infiltration, and reduced regenerative capacity [5,20,21]. These alterations are aggravated by “inflammaging,” a state of chronic low-grade inflammation characterized by increased levels of pro-inflammatory cytokines, which impair anabolic signaling and contribute to sarcopenia progression [22,23,24]. Sedentary behavior further exacerbates this inflammatory state, a concept referred to as “inflamm-inactivity” [10,25]. In this context, biomarkers have emerged as promising tools not only for diagnosing sarcopenia but also for tracking its progression and evaluating responses to interventions [26]. Of particular interest are cytokines, growth factors, and muscle-derived peptides, which may act as both indicators and active mediators of muscle remodeling. Skeletal muscle is now recognized as a secretory organ capable of producing myokines, cytokines specifically released during muscle contraction, that influence systemic physiology [11,27,28]. Building on this concept, the broader term “exerkines” has been introduced to refer to all exercise-induced molecules, regardless of tissue origin, that exert local or systemic effects on health [29,30,31]. These include not only myokines (from muscle), but also hepatokines (from the liver), adipokines (from adipose tissue), metabolites, and extracellular vesicle cargo. Their expression and release are influenced by exercise type, intensity, duration, and by individual factors such as age, sex, and nutritional status [31].

Despite growing interest in this area, the extent to which exercise modulates these molecules in sarcopenic individuals remains unclear. Several trials have explored isolated biomarkers, but a comprehensive synthesis is lacking. This review aims to address this gap by critically examining recent randomized controlled trials (RCTs) investigating exercise-induced changes in circulating biomarkers in sarcopenic populations, with attention to their diagnostic, prognostic, and therapeutic relevance in the context of muscle aging.

## 2. The Effects of Exercise on Biomarkers Related to Sarcopenia: Where We Are

Sarcopenia has increasingly been recognized as a significant public health concern, particularly in the context of population aging. Since 2016, it has been officially classified as a treatable disease with an ICD-10-CM code, highlighting the importance of early diagnosis and targeted interventions [32]. Among non-pharmacological strategies, resistance and multicomponent training are widely recommended as first-line approaches [16,33]. Exercise not only improves physical function and muscle mass but also modulates circulating biomarkers, particularly those related to inflammation, muscle metabolism, and endocrine function [29]. To explore the current evidence, we conducted a structured review of Randomized Controlled Trials (RCTs). We searched PubMed and Google Scholar for studies published between January 2019 and September 2025, using combinations of the following keywords: “sarcopenia”, “exercise”, “physical activity”, “biomarkers”, “myokines”, “exerkines”, “RCT”, and “older adults”. We included studies meeting all of the following criteria: (i) randomized controlled trial design, (ii) presence of a non-exercising control group, and (iii) participants with confirmed sarcopenia. A total of six RCTs met the inclusion criteria and are summarized in Table 1.

The most frequently investigated biomarkers were tumor necrosis factor-alpha (TNF-α) and interleukin-6 (IL-6), each assessed in 57% of studies. Other relevant markers included myostatin (43%), C-reactive protein (CRP) and insulin-like growth factor-1 (IGF-1) (29%), as well as anti-inflammatory or regenerative factors such as interleukin-10 (IL-10), interleukin-15 (IL-15), follistatin, insulin, brain-derived neurotrophic factor (BDNF), and irisin (14%). The average duration of the interventions was 17 weeks (range: 8–24 weeks), with a mean participant age of 69 years (range: 38–84). Resistance training alone was implemented in five out of six studies (83%), while one trial adopted a multicomponent protocol combining resistance, aerobic, and balance training. None of the included RCTs investigated aerobic exercise in isolation. The average dropout rate across studies was 17%, ranging from 0% to 43%. Sample characteristics varied across studies in terms of age range, sex distribution, and baseline functional status, as detailed in Table 1.

The methodological quality of the included studies was evaluated using a domain-based checklist adapted from the PRISMA 2020 statement [40] and the CONSORT 2010 guidelines for randomized controlled trials [41]. Since all included articles were RCTs involving exercise interventions in sarcopenic populations, the criteria were tailored to reflect key methodological domains relevant to this context. Each study was assessed across ten domains: (A) similarity of groups at baseline; (B) reporting of demographic and descriptive characteristics; (C) clearly stated inclusion and exclusion criteria; (D) prespecified primary and secondary outcomes, including how and when they were assessed; (E) reliability or validity of outcome measures; (F) replicable intervention protocols; (G) blinding of outcome assessors; (H) compliance (≥75%); (I) reporting of dropouts (≤30%); and (J) group-specific results with effect sizes and confidence intervals. Each domain was rated as “Yes” (green), “No” (red), “Not Reported” (orange), or “Not Applicable” (grey). Studies were classified as Excellent (9–10 Yes), Good (6–8), Fair (4–5), or Poor (0–3). The results of this appraisal are presented in Table 2.

The mean methodological quality score was 8.3, ranging from 7 to 9. Of the six included studies, four were rated as “Excellent” (score = 9/10) and two as “Good” (score = 7/10), based on the adapted domain-based risk of bias checklist (Table 2).

## 3. Hormones

Growth-related hormones, such as IGF-1, insulin, and growth hormone are fundamental for maintaining skeletal muscle mass, promoting protein synthesis, glucose uptake, and satellite cell activation. However, their circulating levels progressively decline with age, contributing to anabolic resistance and muscle wasting. At the same time, pro-inflammatory cytokines increase, accelerating proteolysis and impairing muscle regeneration [2,42,43,44].

Among these, IGF-1 is one of the most studied anabolic hormones in sarcopenia research. It activates the PI3K/Akt/mTOR signaling pathway, promoting protein synthesis, while concurrently inhibiting catabolic pathways such as FoxO signaling [6,45]. Its muscle-specific isoform, known as mechanical growth factor (MGF), enhances tissue repair and regeneration, particularly in response to mechanical loading [46]. Multiple studies have documented lower IGF-1 and MGF levels in sarcopenic individuals, especially those with sarcopenic obesity, where hormonal dysregulation is accompanied by fat accumulation and metabolic impairment [45,47,48]. Exercise, particularly resistance and combined training, has been shown to stimulate IGF-1 expression and activity, supporting muscle hypertrophy and reducing inflammation. For instance, in the RCT by Ghayomzadeh et al. [34], six months of combined training led to a significant increase in IGF-1 levels. Interestingly, Griffen et al. [35] reported differential effects depending on the training modality, with combined resistance and protein supplementation showing more robust endocrine responses than resistance training alone. These findings underscore the relevance of training type and nutritional context in modulating hormonal responses, with multicomponent protocols potentially offering greater benefits than resistance training alone.

Similarly, insulin plays an essential anabolic role by facilitating glucose uptake, glycogen storage, and amino acid transport into muscle cells [49,50]. However, insulin resistance, common in older adults and individuals with type 2 diabetes, contributes to muscle catabolism and sarcopenic progression. Although insulin levels do not always significantly increase post-intervention, several RCTs, such as those by Griffen et al. [35] and Zhang et al. [39], showed maintenance or stabilization of insulin concentrations in training groups, contrasting with declines in controls. This suggests a protective metabolic effect of exercise, even in the absence of marked hormonal elevation.

## 4. Pro-Inflammatory Biomarkers: Interleukin-6, Tumor Necrosis Factor Alpha and C-Reactive Protein

Chronic low-grade inflammation, or “inflammaging,” plays a central role in sarcopenia progression by promoting muscle catabolism, impairing regeneration, and disrupting anabolic signaling. Among the pro-inflammatory biomarkers most consistently associated with sarcopenia are IL-6, TNF-α, and CRP.

IL-6 is a pleiotropic cytokine produced by multiple cell types, including monocytes, T cells, endothelial cells, and skeletal muscle fibers. Its role is context-dependent: while chronically elevated IL-6 promotes inflammation, protein degradation, and reduced IGF-1 bioavailability, exercise-induced IL-6 has anti-inflammatory and metabolic benefits, such as enhanced glucose uptake, lipolysis, and fat oxidation, primarily via AMPK activation [46,51,52]. Muscle contraction during endurance or resistance training triggers transient increases in IL-6, followed by downstream release of anti-inflammatory cytokines such as IL-10. This is distinct from the persistently elevated IL-6 observed in sarcopenia and metabolic diseases, which activates NF-κB, contributing to muscle atrophy [53]. Among the RCTs included in our review, IL-6 was assessed in four studies, all of which reported reductions following resistance or combined training interventions in older sarcopenic adults [34,35,36,39].

TNF-α is another hallmark of systemic inflammation in aging, known to inhibit the Akt/mTOR pathway, impair myogenesis, and stimulate muscle protein breakdown via the ubiquitin-proteasome system [23,52]. Elevated TNF-α is associated with frailty and poor muscle function in elderly populations [46]. Resistance training protocols have been shown to decrease TNF-α levels, with greater effects observed when exercise is combined with nutritional interventions such as whey protein [35]. For instance, in the RCT by Ghayomzadeh et al. [34], a six-month combined training protocol significantly lowered TNF-α concentrations.

CRP is an acute-phase protein and clinical marker of systemic inflammation often used in both research and clinical settings to evaluate low-grade inflammation. Elevated CRP levels are commonly observed in sarcopenic and frail individuals [23,52]. Although fewer studies have evaluated CRP changes in response to exercise in sarcopenic cohorts, several trials suggest that long-term resistance training may lower CRP, especially when associated with improved body composition and muscle strength. However, among the RCTs analyzed in this review, CRP was assessed in only two studies [34,35], and results were modest and partly inconsistent. Differences in assay sensitivity, baseline inflammation levels, and intervention length may account for this variability.

## 5. Anti-Inflammatory Biomarkers: Interleukin-10 and Interleukin-15

In contrast to pro-inflammatory mediators, anti-inflammatory cytokines play a protective role in muscle health by suppressing chronic inflammation, enhancing regenerative pathways, and delaying the onset of sarcopenia. Two of the most studied in this context are IL-10 and IL-15.

IL-10 is a potent immunomodulatory cytokine that regulates the immune response by inhibiting macrophage activation and suppressing the secretion of TNF-α and IL-6 [30,46,54]. Beyond its anti-inflammatory action, IL-10 facilitates muscle regeneration by shifting macrophage polarization from the pro-inflammatory M1 phenotype to the anti-inflammatory M2 phenotype, a process essential for resolving inflammation and promoting tissue repair. Higher IL-10 levels have been observed in physically active older adults, suggesting a beneficial association with regular exercise [24]. Evidence from RCTs further supports this: in a 24-week resistance training program, Gadelha et al. [36] demonstrated significant increases in IL-10 concentrations in sarcopenic individuals, alongside improvements in body composition and muscle strength. However, IL-10 was assessed in only one of the RCTs included in this review, limiting the generalizability of findings across exercise interventions.

IL-15 is a myokine secreted in response to muscle contraction, with multifunctional roles spanning muscle maintenance, fat metabolism, and immune regulation. Mechanistically, IL-15 promotes myogenesis, suppresses proteolysis, and limits intramuscular fat infiltration by modulating fibro-adipogenic progenitor activity [45]. It also facilitates crosstalk between muscle and adipose tissue, reducing visceral fat and improving insulin sensitivity, largely via AMPK pathway activation. IL-15 levels tend to decline with aging, contributing to the metabolic and structural deterioration seen in sarcopenia. However, Urzi et al. [38] reported significant IL-15 upregulation following a 12-week elastic resistance training protocol in elderly nursing home residents. This suggests that physical activity may restore IL-15 signaling and counteract age-related declines. Nonetheless, IL-15 was also investigated in only one included study, highlighting the need for further trials to confirm its responsiveness to different exercise modalities.

Together, IL-10 and IL-15 illustrate the dual role of exercise as both an anti-inflammatory and anabolic stimulus, reinforcing the concept that regular physical activity can modulate the cytokine environment in favor of muscle preservation, metabolic balance, and healthy aging. Currently, both IL-10 and IL-15 are primarily used in research settings and are not part of routine clinical assessments for sarcopenia. Their implementation as clinical biomarkers would require further validation and standardization.

## 6. Myostatin and Follistatin

Myostatin, also known as growth and differentiation factor 8 (GDF-8), is a potent negative regulator of skeletal muscle mass and function. A member of the transforming growth factor-β (TGF-β) superfamily, myostatin is primarily secreted by skeletal muscle fibers and plays a crucial role in inhibiting myogenesis [6,7,21]. Mechanistically, myostatin binds to activin type IIB receptors (ActRIIB), initiating the Smad2/3 signaling cascade, which suppresses the IGF-1/Akt/mTOR pathway, reduces satellite cell activity, and enhances proteolysis via FoxO1 activation [45,51]. Beyond its direct effects on muscle, myostatin also contributes to metabolic dysfunction by impairing glucose uptake (via inhibition of GLUT4 and AMPK) and promoting inflammation through TNF-α induction [46]. Elevated myostatin levels are consistently observed in individuals with sarcopenia, making it a critical biomarker and a potential therapeutic target. Exercise, particularly resistance and combined training, has been shown to reduce circulating myostatin levels. For instance, Mafi et al. [37] demonstrated significant myostatin downregulation following both resistance training alone and in combination with epicatechin supplementation. Similarly, Griffen et al. [35] observed myostatin modulation in elderly subjects following a 12-week resistance training protocol. However, discrepancies among trials highlight the need for more standardized intervention designs to confirm the extent of exercise-induced effects. Factors such as training modality, exercise duration and intensity, baseline muscle status, and participant characteristics (e.g., age, comorbidities) may partly explain the heterogeneity in results.

In contrast, follistatin is a glycoprotein that binds and neutralizes myostatin, thereby promoting muscle hypertrophy and regeneration. By antagonizing TGF-β family members, follistatin activates Akt/mTOR signaling, enhances protein synthesis, and suppresses muscle degradation [45,55]. It also plays a role in modulating fibro-adipogenic progenitor cells, limiting intramuscular fat deposition and improving muscle quality. Several studies have shown that physical activity upregulates follistatin levels. Endurance and combined training appear particularly effective, though resistance training also yields significant increases, as shown in the RCT by Mafi et al. [37]. These adaptations contribute to improved muscle anabolism and attenuate the effects of aging-related sarcopenia.

The myostatin–follistatin axis thus represents a finely regulated endocrine mechanism responsive to exercise. Its modulation offers promising avenues for biomarker-guided interventions and underscores the molecular basis of physical activity as a therapeutic tool against muscle wasting. At present, however, both myostatin and follistatin are primarily used as research biomarkers, and their implementation in clinical practice remains limited due to the need for further validation and standardization.

## 7. Irisin

Irisin is a myokine produced by the cleavage of the transmembrane protein fibronectin type III domain-containing protein 5 (FNDC5), mainly in response to muscle contraction and regulated by PGC-1α. It plays a pivotal role in the regulation of metabolic homeostasis, mitochondrial biogenesis, and skeletal muscle adaptation [45,46]. Its biological effects include improvements in glucose uptake, oxidative capacity, and muscle regeneration, all of which are particularly relevant in aging-related muscle decline [45]. Mechanistically, irisin activates signaling pathways such as AMPK-PGC1α, IGF-1/Akt/mTOR, and MAPK, which support myogenic differentiation, muscle regeneration, and glucose metabolism, while inducing the browning of white adipose tissue [45,46]. These effects are especially relevant in aging, where plasma irisin levels have been shown to decline with advancing age, correlating with reduced muscle mass, strength, and mitochondrial function. Importantly, several studies have demonstrated that exercise stimulates irisin secretion, with both resistance and endurance training increasing circulating irisin levels in older adults [39,45]. This has led to the proposition of irisin not only as a biomarker of sarcopenia but also as a therapeutic target. Among the randomized controlled trials included in this review, the study by Zhang et al. [39] provides direct evidence of the effects of resistance training on circulating irisin levels in elderly women with sarcopenia. After 12 weeks of elastic resistance training, participants in the intervention group showed a significant increase in irisin compared to baseline and the control group, alongside improvements in muscle strength and physical performance. These findings reinforce the potential of irisin as an exercise-responsive biomarker of muscle health in aging populations. Preclinical studies further support irisin’s protective effects, showing its ability to stimulate satellite cell activation, inhibit FoxO-mediated proteolysis, and improve mitochondrial function. Moreover, reduced irisin levels have been associated with sarcopenic obesity, suggesting a broader role in muscle-fat crosstalk and metabolic regulation [39,46]. Taken together, the evidence highlights irisin as a key exerkine in the context of sarcopenia, with both diagnostic and therapeutic implications. Its modulation through exercise may offer a valuable biomolecular pathway to preserve muscle mass, improve function, and delay the onset of frailty in the elderly. However, despite its potential, irisin is not yet routinely measured in clinical settings, partly due to methodological variability in assay techniques and the need for further standardization.

**Figure 1:**
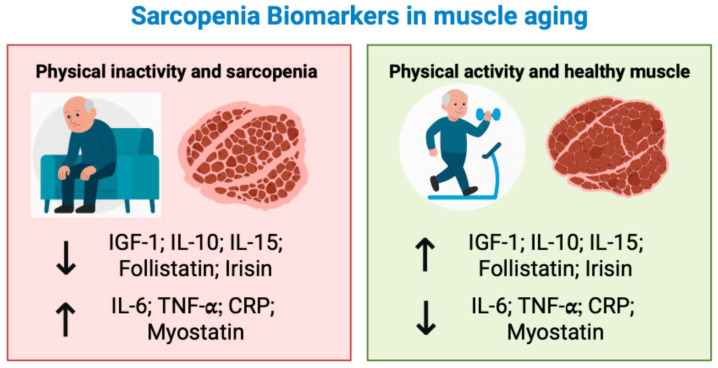
Impact of Physical (In)Activity on Biomarkers and Muscle Health in Older Adults. Schematic representation of the effects of physical activity on selected anabolic and inflammatory biomarkers in older adults. Physical inactivity is associated with an increase in pro-inflammatory and catabolic factors (e.g., IL-6, TNF-α, CRP, myostatin), promoting sarcopenic decline. In contrast, regular exercise stimulates the expression of anabolic and anti-inflammatory mediators (e.g., IGF-1, IL-10, IL-15, follistatin, irisin), contributing to muscle preservation and functional recovery. The upward (↑) and downward (↓) arrows indicate the direction of change in circulating biomarker levels. Specifically, ↑ represents an increase, and ↓ represents a decrease, as influenced by physical activity or inactivity in the context of muscle aging.

Figure 1 illustrates the contrasting biological effects of physical inactivity and regular exercise on key biomarkers implicated in sarcopenia, emphasizing how physical activity fosters an anabolic and anti-inflammatory environment that supports muscle maintenance and regeneration.

## 8. Emerging Biomarkers and Molecular Perspectives

**Figure 2:**
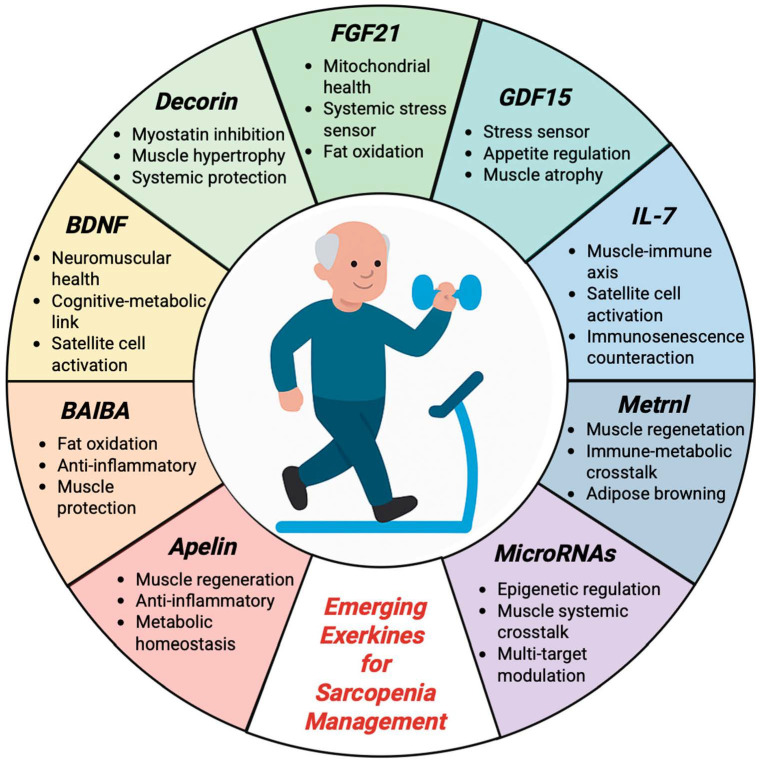
Emerging Exerkines: Key Molecular Mediators of Exercise with Therapeutic Potential in Sarcopenia. Overview of the emerging exercise-responsive biomarkers discussed in Section 8. These molecules, including myokines, cytokines, neurotrophins, and epigenetic regulators, are modulated by physical activity and play key roles in muscle regeneration, metabolic homeostasis, and the pathophysiology of sarcopenia. Each segment corresponds to one of the following biomarkers: Apelin (8.1), Brain-Derived Neutrophic Factor (BAIBA—(8.2), Brain-derived neurotrophic factor (BDNF—8.3), Decorin (8.4), Fibroblast growth factor 21 (FGF21—8.5), Growth differentiation factor 15 (GDF15—8.6), Interleukin 7 (IL-7—8.7), Meteorin-like protein (Metrnl—8.8), and microRNAs (8.9). These factors represent potential diagnostic and therapeutic targets for personalized exercise interventions in aging populations.

Several biomarkers have been associated with skeletal muscle health and sarcopenia and show potential as both diagnostic and therapeutic targets, yet many remain insufficiently explored in the context of physical activity. Further research is needed to clarify their role as exerkines and their relevance in personalized exercise-based interventions against sarcopenia (Figure 2).

### 8.1. Apelin

Apelin is a peptide expressed in various tissues, including skeletal muscle, adipose tissue, heart, and brain, and is secreted by adipocytes. It acts as the endogenous ligand for the APJ receptor, a G protein-coupled receptor widely distributed across multiple organs [30,56]. The apelin/APJ axis regulates a range of physiological processes, including cardiovascular function, metabolic homeostasis, and inflammation [6,45]. In skeletal muscle, apelin promotes protein synthesis and regeneration by stimulating satellite cell proliferation and differentiation [56]. Aging is associated with a marked decline in apelin expression in muscle tissue, contributing to reduced muscle quality and function. This decline has been linked to sarcopenia, particularly in older men, where lower apelin levels correlate with diminished handgrip strength [56]. Animal studies support apelin’s therapeutic potential: in aged mice, apelin supplementation improves muscle mass, enhances regeneration, and reduces inflammation through modulation of macrophage polarization and satellite cell activation [45,46]. In humans, exercise appears to stimulate apelin secretion. Both resistance and endurance training have been shown to elevate apelin levels in muscle and plasma, improving metabolic parameters in obese and diabetic individuals and mitigating the age-related decline of this peptide [45]. However, no RCTs have yet tested this effect in sarcopenic individuals. In summary, apelin is an exercise-responsive molecule studied in both animals and humans, but its clinical applicability in sarcopenia remains under investigation. Future trials should explore its role in exercise programs specifically designed for muscle loss in aging.

### 8.2. Beta-Aminoisobutyric Acid

Beta-aminoisobutyric acid (BAIBA) is a small exercise-induced molecule secreted by skeletal muscle, classified as a myokine with emerging roles in metabolic regulation and inflammation. It exists in two enantiomeric forms (L-BAIBA and D-BAIBA) produced through thymine and valine metabolism, respectively. L-BAIBA is primarily synthesized in skeletal muscle following activation of peroxisome proliferator-activated receptor gamma coactivator-1α (PGC-1α), particularly in response to endurance and resistance exercise [15,57,58]. BAIBA exerts autocrine and paracrine effects, notably enhancing fatty acid oxidation and insulin sensitivity in muscle cells via the AMPK signaling pathway [30,59]. It also contributes to systemic energy homeostasis by promoting the browning of white adipose tissue and reducing overall fat mass. These metabolic adaptations are accompanied by anti-inflammatory effects in both muscle and adipose tissue, reinforcing BAIBA’s role in protecting against metabolic and age-related muscle disorders [45]. To date, most evidence on BAIBA derives from preclinical studies, which suggest that L-BAIBA may help preserve muscle strength and function during aging. However, plasma BAIBA concentrations tend to decline in older adults compared to younger individuals, potentially limiting its protective impact with advancing age. Interestingly, this reduction appears not to be due to impaired muscle secretion of BAIBA, but rather to decreased expression of the Mas-related G protein-coupled receptor type D (MRGPRD), which mediates its biological effects [45]. Although BAIBA shows promise as an exercise-responsive molecule, its translation to clinical settings remains limited. Future research should clarify whether structured physical activity can restore BAIBA signaling in older adults and whether this molecule could serve as a reliable biomarker or therapeutic target in sarcopenia.

### 8.3. Brain-Derived Neutrophic Factor

Brain-derived neurotrophic factor (BDNF) is a member of the neurotrophin family predominantly expressed in the central nervous system, but also synthesized by skeletal muscle. It plays a pivotal role in neuronal survival, synaptic plasticity, and cognitive function, while also contributing to muscle regeneration and metabolic regulation [45,51]. Plasma BDNF levels decline with age and are further reduced in conditions such as neurodegenerative diseases, obesity, type 2 diabetes, and sarcopenia [6,45]. In skeletal muscle, BDNF supports myogenesis and muscle repair by modulating satellite cell activation and proliferation in response to exercise or injury. It also influences muscle fiber composition, favoring a shift toward fast-twitch glycolytic fibers, which are typically more susceptible to age-related atrophy [45]. Physical activity, particularly resistance training, significantly increases both muscle expression and circulating levels of BDNF, with beneficial effects observed in young individuals as well as in older adults and patients with metabolic or neurological disorders [6]. This confirms BDNF as a reliable exercise-responsive biomarker. Importantly, exercise-induced elevations in BDNF may help counteract the neuromuscular decline associated with aging. For instance, BDNF levels are higher in non-frail older women compared to pre-frail individuals, and structured physical therapy has been shown to enhance these levels in both groups [45]. Moreover, in hemodialysis patients, low BDNF concentrations have been linked to reduced physical performance and increased risk of severe sarcopenia and frailty, reinforcing its potential as a biomarker of muscle health [45]. From a therapeutic perspective, BDNF represents a key molecular mediator of the neuro-muscular axis. By promoting motor unit preservation, enhancing fiber regeneration, and supporting neuromuscular adaptations, BDNF emerges as a promising target for interventions aimed at preserving functional capacity during aging. Physical exercise, particularly resistance-based protocols, remains the most effective non-pharmacological strategy to upregulate BDNF and mitigate sarcopenia progression. However, its use as a clinical biomarker remains limited and is currently restricted to research settings.

### 8.4. Decorin

Decorin is a small leucine-rich proteoglycan classified as a myokine, secreted by skeletal muscle during contraction. It plays a central role in regulating muscle mass and function by antagonizing myostatin, a negative regulator of muscle growth [51,53,60]. Through its interaction with the TGF-β family, decorin suppresses the anti-myogenic actions of myostatin and enhances the expression of promyogenic factors such as follistatin, MyoD1, and Mighty, thereby promoting muscle differentiation and hypertrophy [45]. Beyond promoting anabolic signaling, decorin has been shown to prevent muscle atrophy by downregulating muscle-specific ubiquitin ligases such as atrogin-1 and MuRF1, which are key components of proteolytic pathways [45]. Its expression is upregulated following both acute and chronic exercise, particularly resistance training, which concurrently reduces circulating and intramuscular myostatin levels [6,60]. These exercise-induced effects suggest that decorin is a responsive molecule with potential for non-pharmacological muscle preservation. Decorin also exerts pleiotropic effects that extend beyond skeletal muscle. It displays anti-inflammatory, anti-oxidative, anti-fibrotic, and anti-angiotensin activities, contributing to cellular homeostasis and modulating differentiation, proliferation, and apoptosis [6]. These properties highlight its potential role in mitigating both muscular and systemic consequences of aging and chronic diseases. Age-related declines in decorin expression have been observed in human fibroblasts, and experimental models show that decorin deficiency is associated with impaired glucose tolerance, increased visceral adiposity, and altered metabolic profiles [45]. In clinical settings, lower decorin levels have been correlated with reduced muscle mass in patients with liver cirrhosis and were predictive of sarcopenia severity, suggesting its role as a potential biomarker of muscle health [45]. In summary, given its multifaceted role in promoting muscle growth, regulating inflammation, and maintaining metabolic function, decorin emerges as a promising molecular target in the prevention and treatment of sarcopenia. Strategies aimed at enhancing decorin expression through exercise or pharmacological means may offer novel therapeutic avenues to preserve muscle integrity in aging and disease. However, despite these promising findings, decorin is not yet used in routine clinical practice. Current applications remain confined to research settings, and further validation is needed before its implementation as a standard biomarker for sarcopenia.

### 8.5. Fibroblast Growth Factor 21

Fibroblast growth factor 21 (FGF21) is an endocrine member of the FGF family, primarily involved in regulating metabolic homeostasis, inflammation, and muscle physiology. Unlike classical FGFs, FGF21 does not primarily act through mitogenic pathways but rather exerts systemic metabolic effects. It is predominantly produced by the liver, but also secreted by skeletal muscle and adipose tissue, particularly in response to exercise, fasting, and mitochondrial stress [45,46]. In skeletal muscle, FGF21 contributes to mitochondrial function, oxidative metabolism, and myogenic differentiation. It promotes fatty acid oxidation, suppresses lipogenesis, and enhances energy expenditure by inducing the browning of white adipose tissue. Animal models have demonstrated that FGF21 deficiency leads to increased muscle inflammation, elevated expression of atrophy-related genes (e.g., MuRF1, atrogin-1), and reduced AMPK activation. Conversely, FGF21 treatment reverses these changes, supporting its role in muscle preservation and anti-inflammatory regulation [45]. However, evidence in aging humans presents a dualistic role. Elevated circulating FGF21 levels in older adults have been paradoxically associated with decreased muscle mass and strength, potentially reflecting a compensatory response to chronic inflammation, metabolic stress, or mitochondrial dysfunction rather than a direct pathogenic role [61]. This ambiguity suggests that while FGF21 has protective effects in acute metabolic challenges, its chronic elevation may act as a biomarker of systemic distress and sarcopenia progression. Exercise emerges as a potent stimulator of FGF21 expression. Both resistance and endurance training have been shown to increase circulating and muscular FGF21 levels in humans and animals. This upregulation enhances mitochondrial biogenesis, fatty acid oxidation, and anti-inflammatory signaling. In obese mice, progressive resistance training increased FGF21 expression in the soleus muscle, correlating with strength improvements. Similarly, in humans, regular physical activity elevated plasma FGF21 levels, supporting muscle maintenance and lipid metabolism [46]. Preclinical studies involving gene therapy further reinforce FGF21’s therapeutic potential, showing that its overexpression can mitigate age-related weight gain, reduce adipose tissue hypertrophy, and extend lifespan. In summary, FGF21 is an exercise-responsive molecule studied in both animals and humans, with strong mechanistic links to muscle health and metabolic balance. However, its use in clinical practice is still limited, and further validation is needed to determine its utility as a biomarker or therapeutic target in sarcopenia.

### 8.6. Growth Differentiation Factor 15

Growth differentiation factor 15 (GDF15) is a stress-inducible cytokine belonging to the transforming growth factor-β (TGF-β) superfamily. It is secreted by various tissues, including skeletal muscle, liver, and adipose tissue, particularly in response to cellular stress, mitochondrial dysfunction, oxidative damage, and inflammation. Emerging evidence has identified GDF15 as a biomarker closely associated with sarcopenia, as elevated levels correlate with reduced muscle mass, diminished strength, and impaired physical performance in older adults [62]. Mechanistically, GDF15 exerts systemic effects primarily through its interaction with the glial-derived neurotrophic factor receptor alpha-like (GFRAL), a brainstem-specific receptor involved in appetite regulation and energy balance. Chronic overactivation of this axis may contribute to sarcopenia by suppressing appetite and nutrient intake, thereby exacerbating catabolic processes and reducing anabolic support for muscle tissue. At the cellular level, GDF15 has been shown to downregulate protein synthesis and activate protein degradation pathways, including the ubiquitin-proteasome system and autophagy-lysosome machinery, further promoting muscle atrophy. Interestingly, GDF15 exhibits a dual role depending on the physiological context. Acute bouts of exercise transiently elevate circulating GDF15 levels, peaking during recovery phases. This transient increase appears to be part of the adaptive metabolic response, possibly reflecting mitochondrial stress and increased metabolic demand. Kleinert et al. [63] reported that exercise-induced GDF15 likely originates from metabolically active tissues such as the liver rather than skeletal muscle itself. In contrast, chronic elevations in GDF15, as observed in sarcopenic or frail individuals, are indicative of persistent metabolic dysregulation and systemic stress, contributing to progressive muscle wasting. Taken together, these findings highlight GDF15 as an exercise-responsive biomarker with both adaptive and maladaptive roles, depending on the intensity and duration of the physiological stressor. Its unique muscle–brain axis signaling and responsiveness to metabolic stress suggest potential for therapeutic modulation. However, while GDF15 shows promise as a biomarker for sarcopenia monitoring, it is not yet employed in clinical routine. Current evidence is largely preclinical or observational, and further studies are needed to clarify its diagnostic specificity and response to different exercise modalities in aging populations.

### 8.7. Interleukin 7

Interleukin-7 (IL-7) is a pleiotropic cytokine traditionally known for its essential role in lymphocyte development and immune homeostasis. It is primarily produced by stromal cells in lymphoid tissues such as the bone marrow, thymus, spleen, and lymph nodes, but it is also expressed in non-lymphoid tissues including the skin, lungs, intestine, and liver [45,64]. More recently, IL-7 has been identified as a myokine due to its expression and secretion by skeletal muscle cells, particularly in response to physical exercise [45]. In the musculoskeletal system, IL-7 plays a critical role in promoting myogenesis by enhancing satellite cell proliferation and differentiation into mature muscle fibers. This function is especially relevant in the context of muscle injury, adaptation, or age-related muscle loss. However, IL-7 expression decreases with aging, obesity, and high-fat diet intake, factors that also negatively impact muscle regeneration and immune function. Physical activity has been shown to reverse this decline, with both acute and chronic exercise significantly increasing IL-7 levels in muscle and plasma [45]. These findings support IL-7 as an exercise-responsive molecule with potential benefits for both muscle and immune health. Beyond its musculoskeletal effects, IL-7 is essential for immune regulation. It supports T-cell survival and homeostasis, particularly in the maintenance of naïve and memory T-cell populations. This is of particular importance in older adults, in whom immunosenescence contributes to increased susceptibility to infections, chronic inflammation, and impaired muscle repair. In this context, IL-7 appears to bridge muscle and immune health, potentially mitigating both sarcopenia and immune dysfunction through exercise-mediated pathways. Furthermore, emerging evidence suggests that IL-7 may modulate energy metabolism and systemic inflammation, reinforcing its role as a key signaling molecule in the muscle–immune–metabolic axis. However, IL-7 is currently not used in clinical practice and remains a research biomarker. Further studies are needed to confirm its clinical utility and to explore whether targeted exercise programs can effectively harness its therapeutic potential in aging populations.

### 8.8. Meteorin-like Factor

Meteorin-like protein (Metrnl) is a novel myokine secreted predominantly by skeletal muscle and adipose tissue, with emerging roles in muscle regeneration, metabolic regulation, and immune modulation. Its expression is significantly upregulated by stimuli such as cold exposure and physical exercise, leading to increased circulating levels [45,50]. Functionally, Metrnl enhances systemic insulin sensitivity, promotes the browning of white adipose tissue, and increases energy expenditure. Unlike classic adipokines, Metrnl does not directly act on adipocytes but instead exerts its effects via the immune system. It stimulates the activation of macrophages in adipose tissue, promoting an anti-inflammatory phenotype that facilitates thermogenic gene expression and tissue remodeling [65]. These immunometabolic actions are crucial for maintaining metabolic homeostasis and counteracting obesity-associated inflammation and insulin resistance. In skeletal muscle, Metrnl plays a pivotal role in regeneration and repair. Experimental models have shown that Metrnl-deficient mice display impaired muscle healing, with diminished immune cell recruitment and an attenuated transition to anti-inflammatory M2 macrophages. These effects are mediated, at least in part, through a Stat3-dependent pathway that also upregulates IGF-1 expression, thereby enhancing satellite cell activation and myogenesis [45,65]. Moreover, Metrnl supports metabolic health in muscle tissue by activating AMPK and PPARδ signaling, contributing to improved glucose uptake, mitochondrial function, and resistance to inflammation-induced muscle atrophy. Its response to physical activity has been confirmed in both preclinical and human studies, with exercise significantly increasing circulating Metrnl levels. Recent studies in humans have linked higher circulating Metrnl levels to the presence of active brown adipose tissue, suggesting its potential as a biomarker of energy expenditure and metabolic activity [50,60]. Despite these promising findings, Metrnl is not yet adopted in clinical settings. Its current use is limited to research, and further validation is necessary to assess its utility as a biomarker or therapeutic target in sarcopenia. In summary, Metrnl represents a promising exerkine that responds to physical activity and contributes to muscle regeneration and metabolic resilience, but its translational potential remains to be fully explored.

### 8.9. MicroRNAs

MicroRNAs (miRNAs) are small non-coding RNAs that regulate gene expression post-transcriptionally and are increasingly recognized as key modulators of skeletal muscle homeostasis, regeneration, and aging. Their expression is highly dynamic and responds to environmental and physiological stimuli such as exercise, nutritional status, and inflammation. Recent evidence indicates that specific miRNAs are differentially expressed with aging and play a role in the pathophysiology of sarcopenia. For example, miR-181a, miR-434, and miR-221 have been shown to decline with age, correlating with impaired muscle regeneration and increased inflammation, while others such as miR-206 and miR-34a-5p are upregulated and may contribute to altered satellite cell function and senescence [66]. From a mechanistic perspective, miRNAs modulate key pathways involved in muscle adaptation and maintenance, including IGF-1/Akt, SIRT1, and TGF-β signaling. These pathways are intimately involved in mitochondrial function, proteostasis, and satellite cell dynamics, all of which are crucial for preserving muscle integrity in aging [67]. The exercise-induced modulation of miRNAs, such as miR-1, miR-133a, and miR-206, has been linked to improved myogenesis and reduced proteolysis, reinforcing the concept that physical activity can influence muscle phenotype through epigenetic regulation. Beyond skeletal muscle, miRNAs also contribute to the systemic regulation of metabolic and musculoskeletal health. Notably, miR-21-5p and miR-221-3p have been identified as responsive to both physical exercise and dietary intake, acting on pathways such as PI3K/Akt/mTOR and RUNX2, with potential effects on osteogenesis and energy metabolism [68]. These findings extend the relevance of miRNAs beyond muscle tissue and suggest a broader role in coordinating musculoskeletal adaptation. Importantly, circulating miRNAs are detectable in body fluids such as plasma and serum, and their stability makes them attractive candidates for use as non-invasive biomarkers of muscle status and exercise responsiveness. However, while their potential for clinical application is promising, miRNAs are still primarily used in research settings. Future studies are warranted to define standardized panels of exercise-responsive miRNAs and to validate their utility in personalized training programs and early detection of sarcopenia.

## 9. Conclusions

Sarcopenia is a growing health challenge, especially with an aging global population. Its causes are complex, ranging from chronic inflammation to hormonal and metabolic changes, but growing evidence supports the key role of physical exercise in both prevention and treatment. While the condition is still underdiagnosed, new research is shedding light on how exercise interacts with the body at a molecular level.

Among all strategies, resistance training stands out as the most effective way to improve muscle mass, strength, and function in older adults. It works not only by strengthening muscles but also by influencing important biological markers, boosting beneficial molecules like IGF-1, decorin, and BDNF, and lowering harmful ones such as myostatin and TNF-α. Aerobic and combined training add further benefits by improving energy metabolism and reducing inflammation.

These changes are driven by a group of molecules released during exercise, known as exerkines, including irisin, apelin, BAIBA, and IL-15. These molecules do more than reflect physiological status: they actively contribute to muscle repair, fat metabolism, and healthy aging. Understanding and tracking these biomarkers can help personalize training and nutrition plans, making interventions more targeted and effective.

However, to bring this knowledge into everyday clinical or training settings, we need larger and better-designed studies. Future research should focus on standardizing how these biomarkers are measured and integrating them with functional assessments.

In practical terms, combining structured physical activity with smart nutrition, and potentially future therapies targeting myokines, offers the most promising approach. Moving forward, a team-based effort involving clinicians, exercise professionals, and researchers will be crucial.

Despite these promising insights, several limitations still affect current research. Many studies are based on small and heterogeneous samples, often lacking standardized protocols for exercise interventions or biomarker measurement. Furthermore, results are rarely stratified by sex or age, despite evidence that physiological responses to exercise may vary across these factors. This makes it challenging to compare findings across studies or apply them consistently in clinical or athletic settings. Addressing these limitations will be key to validating exercise-responsive biomarkers and moving toward truly personalized approaches to sarcopenia prevention.

The message is clear: staying active is not only safe, but essential to aging well. Exercise is powerful, and science is helping us use it in smarter, more personalized ways to protect muscle health and maintain independence into old age.

## Figures and Tables

**Table 1:** Randomized Control Trials (RCTs) from 2019 to 2025 published on PubMed and Google Scholar that studied the effects of physical activity on myokines related to sarcopenia.

Author	Participants	Age Mean ± SD	Intervention	Results		
				Drop-Out	Sarcopenia	Biomarkers
Ghayomzadeh et al. [34]	40 subjects with HIV infection (20 men and 20 women) randomized in: *combined training* (n = 20; men = 10, women = 10), *no exercise* (n = 20; men = 10, women = 10)	38 ± 7	*Duration*: 6 months *Frequency*: 3 session/week *Exercise Combined*: -*Endurance*: 20 min on treadmill at 65–80% of THR -*Resistance*: 3 × 4–20 at 50/85%	3 subjects (2 in combined training and 1 in no exercise)	*Combined training:* ↑ ALMI ^a,b^, ↑ Handgrip test ^a,b^, ↑ TUG ^a,b^, ↓ FM ^a^, ↑ FFM ^a,b^ *No exercise*: ↓ ALMI ^b^, ↓ Handgrip test ^b^, ↓ TUG ^b^ ↑ FM ^b^, ↓ FFM ^b^	*Combined training*: ↓ IL-6 ^a,b^, ↓ TNF-α ^a,b^, ↑ IGF-1 ^a,b^, ↓ myostatin ^a,b^ *No exercise*: ↑ IL-6 ^a,b^, ↑ TNF-α ^a,b^, ↑ IGF-1 ^b^, ↓ myostatin ^b^
Griffen et al. [35]	39 subjects randomized in: *control* (CON; n = 10), *whey protein* (PRO; n = 10), *resistance exercise* (EX + CON; n = 10), *resistance exercise + whey protein* (EX + PRO; n = 9)	67 ± 1	*Duration*: 12 weeks *Frequency*: 2 session/week *Exercise*: 3 × 10–12 from 60% to 80% of 1 RM	3 subjects (1 in CON, 1 in PRO, 1 in EX + CON)	*CON:* No changes *PRO:* ↑ 4 m gait speed *EX* + *CON*: ↑ leg extension ^a,b^, ↑ leg press ^a,b^ *EX* + *PRO:* ↑ leg extension ^a,b^, ↑ leg press ^a,b^, ↓ Waist circumference ^b^ *CON* + *PRO*: ↑ FM ^b^, ↑ BMI ^b^ *EX* + *CON* + *EX* + *PRO:* ↑ FFM ^b^, ↓ FM ^a^, ↑ 6MWT ^a^	*CON*: No changes *PRO*: No changes *EX + CON*: ↓ insulin ^a^, ↓ IL-6, ↓ TNF-α *EX* + *PRO*: ↓ IL-6 ^a^, ↓ TNF-α ^a^, ↑ salivary cortisol *PRO* + *EX* + *PRO*: ↑ salivary cortisol ^a^, ↑ myostatin ^a^
Gadelha et al. [36]	137 subjects with chronic kidney disease randomized in: *Resistance Training* (n = 72) and *Control* (n = 67)	64 ± 4	*Duration*: 24 weeks *Frequency*: 3 session/week *Exercise*: bodyweight, e-Lastic cable, Bumbbells, Weighted cuffs	32 subjects (17 in control and 15 in resistance training)	*Resistance Training*: ↓ Waist Circunference ^c^, ↓ FM ^c^, ↑ Handgrip Strength ^c^, ↓ TUG ^c^, ↑ 6MWT ^c^, *Control:* No Changes	*Resistance Training*: ↓ IL-6, ↓ TNF-α ^c^, ↑ IL-10 *Control*: No Changes
Mafi et al. [37]	68 elderly males randomized in: *Resistance Training* (RT, n = 17), *Resistance Training* + *Epicatechin* (RT + EP, n = 17), *Epicatechin* (EP, n = 17), *Placebo* (PL, n = 17)	69 ± 3	*Duration*: 8 weeks *Frequency*: 3 session/week *Exercise*: 45 min of resistance exercise [3 × 8–12 (60–80% 1 RM)]	6 subjects (3 in RT, 2 in RT + EP, 1 in PL)	*Resistance Training*: ↑ BMI, ↑ ASMMI, ↑ leg press, ↑ chest press, ↑ TUG *Resistance Training* + *Epicatechin*: ↑ BMI, ↑ ASMMI, ↑ leg press, ↑ chest press, ↑ TUG *Epicatechin*: ↑ ASMMI, ↑ TUG, *Placebo*: No changes	*Resistance Training*: ↓ Myostatin, ↑ Follistatin *Resistance Training* + *Epicatechin*: ↓ Myostatin, ↑ Follistatin *Epicatechin*: ↑ Follistatin *Placebo*: No changes
Urzi et al. [38]	35 female nursing home residents randomized in: *training group* (n = 18) and *control group* (n = 17)	84 ± 8	*Duration*: 12 weeks *Frequency*: 3 session/week *Exercise*: 40 min of progressive elastic resistance training program	15 subjects (7 in training group and 8 in control group)	*Training Group*: ↑ SSPB ^a^, ↑ Gait Speed ^a^, ↑ Chair rise ^d^ *Control Group*: No changes	Training Group: ↑ IL-15, ↑ BDNF ^a^ *Control Group*: No changes
Zhang et al. [39]	60 older adults with sarcopenia randomized in: multicomponent exercise group (n = 30) and control group (n = 30)	72 ± 6	*Duration*: 12 weeks *Frequency*: 3 session/week *Exercise*: multicomponent training including resistance, balance, mobility, and walking (60 min/session)	4 subjects (2 in exercise group and 2 in control group	*Exercise group*: ↑ ASMMI ^a,b^, ↑ Handgrip strength ^a,b^, ↑ SPPB ^a,b^, ↓ TUG ^a,b^ *Control group*: No changes	*Exercise group*: ↑ Irisin ^a,b^ *Control group*: No changes

Legend: HIV = Human Immunodeficiency Virus; ALMI = Appendicular Lean Mass Index; TUG = Time Up and Go test; THR = age-predicted target heart rate; FM = Fat Mass; FFM = Fat Free Mass; 6MWT = 6 Minutes Walking Test; N.A. = Not Applicable; ASMMI = Appendicular Skeletal Muscle Mass Index; SPPB = Short Physical Performance Battery; IL-6 = Interleukin-6; TNF-α = Tumor necrosis factor-alpha; CRP = C-reactive protein; IGF-1 = Insulin-like growth factor-1; IL-10 = Interleukin-10; IL-15 = Interleukin-15; BDNF = Brain-derived neurotrophic factor; ↑ = Increase after exercise; ↓ = Decrease after exercise. ^a^ Significant difference compared with baseline values *p* < 0.05; ^b^ Significant Group × time interaction; ^c^ Significant difference comparted with Control values *p* < 0.05; ^d^ Significant difference compared with baseline values *p* < 0.001.

**Table 2:** Risk of bias assessment of the included randomized controlled trials (A–J domains).

Reference	A	B	C	D	E	F	G	H	I	J	Quality Score	Evaluation
Ghayomzadeh et al. [34]											9/10	Excellent
Griffen et al. [35]											9/10	Excellent
Gadelha et al. [36]											9/10	Excellent
Mafi et al. [37]											7/10	Good
Urzi et al. [38]											7/10	Good
Zhang et al. [39]											9/10	Excellent

Legend. A: groups at baseline were similar (group size, sex, age < 10% difference); B: baseline descriptive characteristics for each group; C: inclusion and/or exclusion criteria for subjects; D: defined prespecified primary and secondary outcome measures, including how and when they were assessed; E: investigated reliability or validity of outcome measures; F: sufficiently described treatment protocol (replicable); G: outcome assessors blinded (where applicable); H: acceptable compliance (≥75%); I: reported dropouts (maximum ≤ 30%); J: group-specific results and effect size reported (e.g., mean, SD, CI). Color Code: Green = Yes, Orange = Not reported.

## Data Availability

No new data were created or analyzed in this study. Data sharing is not applicable to this article.

## Abbreviations

1 RM	One Repetition Maximum
6MWT	Six-Minute Walk Test
ALMI	Appendicular Lean Mass Index
AMPK	AMP-Activated Protein Kinase
ASMMI	Appendicular Skeletal Muscle Mass Index
BAIBA	Beta-Aminoisobutyric Acid
BDNF	Brain-Derived Neurotrophic Factor
BMI	Body Mass Index
CON	Control Group
CRP	C-Reactive Protein
DEXA	Dual-Energy X-ray Absorptiometry
EX	Exercise
EX + CON	Exercise + Control Group
EX + PRO	Exercise + Protein Supplement Group
FFM	Fat-Free Mass
FM	Fat Mass
FNDC5	Fibronectin Type III Domain-Containing Protein 5
FoxO	Forkhead Box O
FGF21	Fibroblast Growth Factor 21
GDF15	Growth Differentiation Factor 15
GDF-8	Growth Differentiation Factor 8 (Myostatin)
GLUT4	Glucose Transporter Type 4
GFRAL	Glial Cell Line-Derived Neurotrophic Factor Family Receptor Alpha-Like
IGF-1	Insulin-Like Growth Factor 1
IL	Interleukin
MGF	Mechanical Growth Factor
Metrnl	Meteorin-Like Protein
mTOR	Mammalian Target of Rapamycin
MuRF1	Muscle RING Finger 1
NF-κB	Nuclear Factor Kappa-Light-Chain-Enhancer of Activated B Cells
PL	Placebo
PI3K	Phosphoinositide 3-Kinase
PRO	Protein Supplement Group
PGC-1α	Peroxisome Proliferator-Activated Receptor Gamma Coactivator 1-Alpha
RT	Resistance Training
RT + EP	Resistance Training + Epicatechin
SD	Standard Deviation
SSPB	Short Physical Performance Battery
T2D	Type 2 Diabetes
THR	Target Heart Rate
TNF-α	Tumor Necrosis Factor Alpha
TUG	Timed Up and Go Test
